# Headache in transient ischemic attacks

**DOI:** 10.1186/s10194-018-0888-5

**Published:** 2018-07-27

**Authors:** Elena R. Lebedeva, Natalia M. Gurary, Jes Olesen

**Affiliations:** 1 Department of Neurology and Neurosurgery, The Ural State Medical University, Repina 3, Yekaterinburg, 620028 Russia; 2 International Headache Center “Europe-Asia”, Yekaterinburg, Russia; 3 Medical Union “New Hospital”, Yekaterinburg, Russia; 4 Danish Headache Center, Department of Neurology, Rigshospitalet-Glostrup, University of Copenhagen, Copenhagen, Denmark

**Keywords:** Transient ischemic attack, TIA, Secondary headache disorders, Migraine, Warning headache

## Abstract

**Background:**

Headache is a common feature in acute cerebrovascular disease but no studies have evaluated the prevalence of specific headache types in patients with transient ischemic attacks (TIA). The purpose of the present study was to analyze all headaches within the last year and the last week before TIA and at the time of TIA.

**Methods:**

Eligible patients with TIA (*n* = 120, mean age 56.1, females 55%) had focal brain or retinal ischemia with resolution of symptoms within 24 h without presence of new infarction on MRI with DWI (*n* = 112) or CT (*n* = 8). All patients were evaluated within one day of admission by a single neurologist. As a control group we used patients (*n* = 192, mean age 58.7, females 64%) admitted with diagnoses “lumbago”, “lumbar spine osteochondrosis” or “gastrointestinal ulcer”.

**Results:**

One-year prevalence of migraine without aura was significantly higher in TIA patients than in controls: 20.8% and 7.8% respectively (*p* = 0.002, OR 3.1, 95% CI 1.6–6.2). 22 patients (18.3%) had sentinel or warning headache within the last week before TIA. At the time of TIA a new type of headache was observed in 16 patients (13.3%). No controls had a new type of headache. 12 of these 16 patients had migraine-like headache, 8 patients had tension-type-like headache and one patient thunderclap headache. Posterior circulation TIA was associated with headaches within last week before TIA and at the time of TIA much more frequently than anterior circulation TIA.

**Conclusions:**

The one year prevalence of migraine was significantly higher in TIA patients than in controls and so was the prevalence of headache within the last week before TIA and at the time of TIA. A previous headache that worsens and a new type of headache can be a warning of impending TIA.

## Background

Several previous studies have shown that headache is a common feature in TIA, its frequency varies from 16% till 36% in different studies [1–10]. These studies have mostly included all kinds of CVD with a minority of transient ischemic attacks (TIA). No recent studies have specifically evaluated the prevalence of headache in patients with TIA and none have diagnosed previous headaches and headaches at the time of TIA according to internationally accepted diagnostic criteria [11].

For a detailed analysis of headaches in TIA it is necessary to characterize not only the headache occurring at the time of TIA but also previous headaches such as migraine and tension-type headache. Otherwise it is impossible to distinguish between an attack of usual headache and a new type of headache occurring at the time of TIA. In order to make this distinction, extensive semi-structured neurologically conducted interviews are necessary, and they should be done as soon as possible after TIA. To the best of our knowledge no such study has ever been performed.

Here we present a prospective study of 120 consecutive patients with TIA using a validated extensive semistructured professionally conducted interview done as soon as the TIA diagnosis including neuroimaging had been secured after admission. The aims of this study were to analyze all headaches within the last year before TIA, all headaches within the last week before TIA (sentinel headache) and all headaches occurring within 24 h after TIA. Our hypothesis was that TIA may cause headaches and that headache may be a warning sign of impending TIA.

## Methods

### Study populations

The period of recruitment of patients was from April 2014 till May 2016. The study population was admitted to the stroke unit of city hospital “New Hospital” in Yekaterinburg, Russia. Inclusion criteria were focal brain or retinal ischemia with resolution of symptoms within 24 h and without presence of new infarction on magnetic resonance imaging (MRI) with diffusion weighted imaging (DWI) or *computed tomography*(CT). Excluded were patients with previous cerebrovascular disease or other serious neurological disease and memory problems. All patients were evaluated within one day of admission, usually within a few hours by a neurologist. 62 patients had TIA < 6 h before admission to the hospital, 50 patients had TIA in interval from 6 to 12 h, 6 had TIA in interval from 13 to 24 h and two had TIA in interval from 24 to 36 h before admission.

A total of 131 patients were examined, 11 patients were excluded because most of them had difficulties to recall essential information and memory problems. 112 patients had MRI with DWI and 8 had CT. These examinations were done immediately after admission to the hospital. All TIA cases were subdivided into anterior and posterior circulation TIA.

As a control group we used patients who were admitted to the emergency room without acute neurological deficits or serious neurological or somatic disorders. We examined 225 controls.33 patients were excluded and 192 patients were included.

### Evaluation

One neurologist (N.M.G.) collected patient data prospectively, using a standardized case-report form during face-to-face interviews during admission to the emergency room in controls. Information about patients, medical history and risk factors imaging and laboratory tests were recorded. We recorded history of headache during last year, during 1 week before TIA and at the time of TIA defined as within 24 h of TIA onset. We used extensive semi-structured interview forms that contained all necessary information to diagnose previous headaches.

### Definitions and diagnostic criteria

TIA was defined as a transient episode of neurological dysfunction caused by focal brain, spinal cord, or retinal ischemia, without acute infarction [12].

The diagnoses of previous and present headaches were made according to the explicit diagnostic criteria of the International Headache Society, the International Classification of Headache Disorders ICHD-3 [11]. We recorded headache within the last year and last week before TIA and within 24 h after onset of TIA. We distinguished between previous headache without change of characteristics, headache with change of characteristics and new type of headache. We defined a new type of headache in TIA as a headache which arose for the first time in the week before or within 24 h after onset of TIA. Migraine or tension type headache with changes of characteristics within the last week before TIA or within 24 h after onset of TIA as well as migraine or tension type headache as a new type of headache were defined as migraine-like headache and tension- type-like headache respectively. We did not apply the diagnostic criteria for “headache attributed to TIA” of the ICHD-3 because the purpose of the present study was to compare all kinds of headaches in patients with TIA to a matched control group.

### Ethical considerations

The Medical Ethics Committee of the Urals State Medical University approved this study. All respondents were informed of the purpose of the survey. Written informed consent was obtained from all participants.

### Statistical analysis

Statistical analyses were performed with SPSS 17.0 software. Continuous variables were summarized as means, and categorical variables as numbers and percentages. We used chi-squared to compare distributions of categorical variables between groups. We set statistical significance at *P* < 0.05. We first used binary logistic regression to estimate odds ratios (OR) with 95% confidence intervals (CI) for consulting and laboratory data. Prevalence estimates and 95% CI for the prevalence estimates of migraine and other headache were determined using previously described methods [13, 14].

## Results

### Characteristics of patients with TIA and controls

Table 1 shows the distribution of patients with TIA and controls by age and sex. The mean age of patients with TIA and controls did not differ significantly: 56.1 and 58.7 respectively. Females prevailed in the control group (64%). Control patients were admitted to the emergency room with the following diagnoses: “lumbago” or “lumbar spine osteochondrosis” (*n* = 99), “pancreatitis” (*n*= 62), “gastrointestinal ulcer” (*n* = 7), tick bite (*n* = 14), irritable bowel syndrome (*n* = 2), paroxysmal benign positional vertigo (*n* = 2), arthritis (*n* = 5), allergic reaction (*n* = 1). Most patients (106 patients, 88%) had TIA in the anterior circulation system and only few (14 patients, 12%) in the posterior circulation system. Seven patients (5.8%) had two or more attacks of TIA. Table 2 shows clinical characteristics of patients with TIA and controls. The prevalence of the following factors was significantly higher in TIA patients than in controls: consumption of strong alcoholic beverages, arterial hypertension, atrial fibrillation, low physical activity, family history of stroke in first degree relatives, hypercholesterolemia, angina pectoris.

**Table 1:** Distribution of patients with TIA and controls by age and sex

Sex	Age interval							
	15–25	26–35	36–45	46–55	56–65	66–75	76–90	Mean age
Male TIA patinets (*n* = 55)	3 (5.5%)	4 (7.3%)	5 (9.1%)	7 (12.7%)	22 (40%)	10 (18.2%)	4 (7.3%)	56.5
Male controls (*n* = 69)	0 (0.0%)	2 (2.9%)	11 (15.9%)	24 (34.8%)	17 (24.6%)	9 (13.0%)	6 (8.7%)	56.5
Female TIA patients (*n* = 65)	5 (7.7%)	7 (10.8%)	7 (10.8%)	9 (13.8%)	16 (24.6%)	11 (16.9%)	10 (15.4%)	55.7
Female controls (*n* = 123)	0 (0.0%)	5 (4.1%)	11 (8.9%)	25 (20.3%)	41 (33.3%)	26 (21.1%)	15 (12.2%)	59.9
All TIA patients (*n* = 120)	8 (6.7%)	11 (9.2%)	12 (10.0%)	16 (13.3%)	38 (31.7%)	21 (17.5%)	14 (11.7%)	56,1
All control patients (*n* = 192)	0 (0.0%)	7 (3.6%)	22 (11.5%)	49 (25.5%)	58 (30.2%)	35 (18.2%)	21 (10.9%)	58.7

**Table 2:** Clinical characteristics of patients with TIA and controls

Characteristics	Patients with TIA (*n* = 120)	Controls (*n* = 192)	*Р* value
Mean age	56.1	58.7	0.1
Male, n (%)	55 (45.8%)	69 (35.9%)	0.1
Current smoker, n (%)	38 (31.7%)	45 (23.4%)	0.1
Consumption of light alcoholic beverages, n (%)	5 (4.2%)	11 (5.7%)	0.7
Consumption of strong alcoholic beverages, n (%)	23 (19.2%)	18 (9.4%)	0.02
Arterial hypertension, n (%)	94 (78.3%)	108 (56.3%)	< 0.001
Diabetes mellitus, n (%)	10 (8.3%)	14 (7.3%)	0.9
Hyperglycemia n (%)	23 (19.2%)	26 (13.5%)	0.2
Atrial fibrillation, n (%)	14 (11.6%)	7 (3.6%)	0.01
Body mass index > 25, n (%)	68 (56.7%)	118 (61.5%)	0.5
Low physical activity, n (%)	45 (37.5%)	28 (14.6%)	< 0.001
Family history of stroke in first degree relatives, n (%)	53 (44.2%)	48(25.0%)	0.001
Peripheral artery disease, n (%)	2 (1.7%)	1 (0.5%)	0.7
Hypercholesterolemia, n (%)	56 (46.7%)	55 (28.6%)	0.002
Angina pectoris, n (%)	32 (26.7%)	18 (9.4%)	< 0.001
Myocardial infarction, n (%)	11 (9.2%)	7 (3.6%)	0.07

The duration of TIA varied from 5 min to 24 h. 42 patients (35%) had resolution of all symptoms within 60 min. Among them 22 patients (18,3%) had duration of TIA from 2 till 15 min and 20 patients (16,7%) 16–60 min. 18 patients (15%) had duration of TIA from 1 h to 3 h and 60 patients (50%) had duration from 3 h to 24 h. 86 patients with TIA had sensory deficits, 68 patients had speech disturbances, 73 patients had unilateral motor weakness [14].

All patients with TIA underwent a stroke risk assessment according to the ABCD2 score. A low risk of stroke (0–3 points) was registered in 62 patients (51.6%), moderate risk (4–5 points) in 54 patients (45%), high risk (6–7 points) in 4 patients (3.3%).

### Headache within last year (excepting last week) before TIA

The prevalence of all primary headache disorders in patients with TIA during the last year (excepting the last week) before TIA and in controls is presented in Table 3. Ninety patients (75%) had tension-type headache (TTH) and 27 patients (22.5%) had migraine during 1 year before TIA. Most patients with migraine also had TTH. 20 patients had no headache at all. The prevalence of chronic headaches was 4%. These patients all had TTH and/or migraine and were thus included in the above numbers. Only the one-year prevalence of migraine without aura was significantly higher in patients with TIA than in controls: 20.8% and 7.8% respectively (*p* = 0.002, OR 3.1, 95% CI 1.6–6.2). This was also true for females only: 33.8% and 11.4% respectively (*p* < 0.001, OR 4.0, 95% CI 1.9–8.5).

**Table 3:** One-year prevalence^a^ of primary headache disorders in patients with TIA (*n* = 120) and in controls (*n* = 192) according ICHD 3

Type of headaches	Males with TIA (*n* = 55)	Male controls (*n* = 69)	Р, ОR (95% CI)	Females with TIA (*n* = 65)	Female controls (*n* = 123)	Р, ОR (95% CI)	All patients with TIA (*n* = 120)	All controls (*n* = 192)	Р, ОR (95% CI)
Migraine without aura	3 (5.5%)^b^	1 (1.4%)	0.5	22 (33.8%)	14 (11.4%)	< 0.001, 4.0; 1.9–8.5	25 (20.8%)	15 (7.8%)	0.002, 3.1; 1.6–6.2
Migraine with aura	0	0	–	1 (1.5%)	1 (0.8%)	0.8	1 (0.8%)	1 (0.52%)	0.7
Chronic migraine	0	0	–	1 (1.5%)	1 (0.8%)	0.8	1 (0.8%)	1 (0.52%)	0.7
Episodic TTH^c^	40 (72.7%)	48 (69%)	0.9	46 (70.8%)	84 (68.3%)	0.9	86 (71.7%)	132 (68.7%)	0.6
Chronic TTH	0	0	–	4 (6.2%)	3 (2.4%)	0.4	4 (3.3%)	3 (1.6%)	0.5
Cluster headache	1 (1.8%)	0	0.9	0	1 (0.81%)	0.7	1 (0.8%)	1 (0.52%)	0.7
Medication overuse headache (analgesics)	0	0	–	2 (3.1%)	0	0.2	2 (1.7%)	0	0.3
Absence of headache	11 (20%)	20 (29%)	0.3	9 (13.8%)	14 (11.4%)	0.8	20 (16.6%)	34 (17.7%)	0.9

^a^Prevalence of primary headache disorders in patients with TIA were calculated during last year excepting the last week before TIA

^b^Percentages in this table were calculated using number of patients with TIA and controls (males, females, all) indicated in upper row of the table

^c^TTH – tension type of headache

### Headache within the last week before TIA

These headaches and headaches in controls are described in Table 4. 26 of 120 patients with TIA (21.6%) had headache within the last week before TIA versus 12 of 192 of controls (6.2%) (*p* < 0.01). All patients (*n* = 14, 100%)with posterior circulation TIA and 12 of 106 patients (11.3%) with anterior circulation TIA had headache in this period. Headache occurred most often within one day before TIA (15 out of 26 patients, 57.6%), 11 out of 26 patients (42.3%) had headache 2–7 days before TIA.

**Table 4:** Headache in patients during 1 week before TIA (*n* = 120) and during last week before interview in controls (*n* = 192)

Type of headache	Previous headache without changes in patients with TIA	Previous headache without changes in controls	Р, ОR (95% CI)	Previous headache with changes in patients with TIA	Previous headache with changes in controls	Р, ОR (95% CI)	New type of headache in patients with TIA	New type of headache in controls	Р, ОR (95% CI)
Migraine without aura^a^	2(1.6%)	0 (0%)	0.07	4 (3.3%)	0	0.01	2 (1.6%)	0	0.07
Migraine with aura^a^	0	0	–	0	0	–	1 (0.8%)	0	0.2
TTH^a^	2 (1.6%)	10 (5.2%)	0.1	12 (10%)	2 (1.0%)	0.0002	2 (1.6%)	0	0.01
Cluster headache	0	0	–	0	0	–	0	0	–
Thunderclap headache	N/A^b^	N/A	–	N/A		–	1 (0.8%)	0	0.2
All headaches	4 (3.3%)	10 (5.2%)	0.4	16 (13.3%)	2 (1.0%)	0.00001	6 (5%)	0	0.0003

^a^Here and in the Table 5: migraine with and without aura with changes of characteristics and tension type headache with changes of characteristics as well as migraine with and without aura or tension type headache as a new type of headache were defined as migraine-like headache and tension type-like headache respectively in the text of the article because they were probably attributed to TIA

^b^Here and in the Table 5: N/A not applicable

Four patients (3.3%) had previous headache without changes of characteristics within a week of TIA and 10 controls (5.2%).Seven patients (5.8%) and no controls had migraine-like headaches within the last week before TIA. Patients with posterior circulation TIA had migraine-like headache more often (6 of 14, 42.8%) than patients with anterior circulation TIA (1 of 106, 0.9%). Four of 7 patients had a past history of migraine during the last year. Before TIA their migraine attacks became daily and stronger than usual. In three patients the migraine-like attacks appeared for the first time. All these seven patients could have sentinel or warning headache.

Fourteen patients (11.6%) had tension-type-like headache during the last week before TIA and 2 controls (1.0%). The percentage of patients with posterior circulation TIA with such headaches was much greater (57.1%) than the percentage with anterior circulation TIA (7.5%).12 of these patients had a past history of headache. They had increases in duration, frequency, and/or intensity of their previous headaches. The prevalence of TTH with changes of characteristics was significantly higher in TIA patients (10%) than in controls (1.0%). Two patients with TIA had a new tension-type- like headache and no controls. All these 14 patients with tension-type-like headache could have sentinel or warning headache.

Totally 22 patients (14 with tension-type-like headache, 7 with migraine-like headache and one with thunderclap headache) had sentinel or warning headache within the last week before TIA.

### Headache at the time of TIA

A new type of headache was observed in 16 patients (13.3%) (Table 5). No controls had a new type of headache. 12 of the 16patients had migraine-like headache, 8 patients had tension-type-like headache and one patient thunderclap headache. Among the sixteen patients, 12 had posterior circulation TIA (86% of patients with posterior circulation TIA) and 4 had anterior circulation TIA (3.7% of patients with anterior circulation TIA). There was no relation between duration of TIA symptoms and occurrence of a new type of headache. 11 patients with a new type of headache (69%) were older than 45 years.

**Table 5:** Headaches at the time of TIA onset (*n* = 120) and at admission of controls (*n* = 192)

Type of headache	Headaches at the time of development of TIA								
	Previous headache without changes in patients with TIA	Previous headache without changes in controls	Р, ОR (95% CI)	Previous headache with changes in patients with TIA	Previous headache with changes in controls	Р, ОR (95% CI)	New type of headache in patients with TIA	New type of headache in patients with controls	Р, ОR (95% CI)
Migraine without aura	2 (1.6%)	5 (4.1%)	0.6	4 (3.3%)	0	0.01	11 (9.1%)	0	0.00001
Migraine with aura	0	0	–	0	0	–	1 (0.83%)	0	0.2
TTH	6 (5%)	4 (3.3%)	0.1	5 (4.1%)	0	0.004	3 (2.5%)	0	0.02
Cluster headache	0	0	–	0	0	–	0	0	0
Thunderclap headache	N/A	N/A	–	N/A	N/A	–	1 (0.83%)	0	0.2
All headaches	8 (6.6%)	9 (4.6%)	0.4	9 (7.5%)	0	0.0001	16 (13.3%)	0	0.00001

A previous headache with changed characteristics was found in 9 of 120 patients (7.5%) and no in controls. Four of these patients had migraine-like headache and 5 tension-type-like headache. Six patients had TIA in the posterior circulation (42.8%) and 3 in the anterior circulation (2.8%). Four patients had a past history of migraine without aura and 5 a history of TTH. They had increased intensity, frequency or altered localization or they became refractory to usual treatment Seven patients had headache at the same time as TIA and two after TIA. Six patients had duration of TIA more than 3 h and three patients had duration 10–15 min.

Eight of 120 patients (6.6%) and 9 controls (4.6%) had a usual headache without any changes. Four patients had TIA in the posterior circulation and the remaining four in the anterior circulation. Six patients had headache at the same time as TIA and two after TIA. Six patients had duration of TIA more than 3 h and two patients had duration 15–30 min.

We performed follow-up of 118 out of 120 patients with TIA, the average follow-up period was 30 months. Four patients reported repeated TIA. Ischemic strokes happened in 7 patients. We found only one case of migraine with aura which was missed during the first interview.

## Discussion

The major findings of the present study were that TIA patients compared to controls more frequently had migraine within the previous year, more often had headache within one week before TIA and at the time of TIA.

### Previous studies of headache in TIA

Only few studies have attempted to characterize headache in patients with TIA and no studies were performed in the past 10 years [1–10]. In most studies headaches were analyzed in TIA patients together with stroke patients [1, 2, 4, 9] but two studies analyzed TIA patients separately [5, 10]. Patients were asked in one study about the presence and localization of headache at symptom onset and to describe the quality of headache according to predefined categories: dull, pressing, stabbing, burning, pulsate, or circular [2]. In the other study patients were asked about the presence and nature (throbbing versus constant) of headache [4]. In three other studies patients were asked about onset, duration, location and quality of headache [1, 5, 9, 10]. Patients were asked about headache prior to TIA only in two studies [1, 5]. All previous studies used CT but not MRI with DWI for detection of infarct and many studies therefore may have included small infarcts. Besides no study could make a specific diagnosis of headache because of absence of classification that time or lack of information about necessary characteristics of the headache. The few studies with a big number of TIA patients did not use a detailed interview about previous and current headache [2, 3]. Therefore, it is difficult to compare our results with previous studies. The character of headaches at TIA onset was different in different studies: throbbing [1, 9] or diffuse [5] or generalized non-localized [10]. The overall prevalence of headache at the time of TIA varied from 16% to 36% and is thus in accordance with our results [1–10].

### Methodological considerations

Several principles should be taken into account in studies of headache in TIA patients. First of all, it is impossible to know the exact diagnosis of headache without using the diagnostic criteria of the International classification of headache [11]. This requires a professional semi-structured interview about previous and current headache, preferably face to face, because some important characteristics of headache can otherwise be missed in acutely ill patients. It is also necessary to record the exact timing of headache and TIA and to use a generally accepted definition of transient ischemic attacks including MRI with DWI to exclude acute infarcts.

Headache can only manifest in a limited number of ways. Thus, most secondary headaches including headache in TIA patients have the characteristics of tension-type headache or migraine without aura. If there is a close temporal relation, it must, however, be classified as a secondary headache attributed to the causative disorder according to ICHD-3 [11]. Since most of the headaches encountered in the present study were new or had altered characteristics and occurred significantly more often than in the control group, we have chosen to call those occurring in the week before or at the time of TIA migraine–like headaches and tension-type-like headaches.

### Significance of our findings

Our results have important clinical implications and also bespeak to some extent the possible mechanisms of headache and migraine.

#### Can headache be a warning sign of TIA?

From transcranial doppler monitoring it is well known that patients have many neurologically silent cerebral emboli [15]. Perhaps some of them cause headache. Our study showed beyond doubts that headache, more specifically migraine-like headache, is more common within the previous year before TIA than in controls. The difference was even more pronounced within one week of TIA and particularly within the last day before TIA. It seems overwhelmingly likely, therefore, that headache, especially headache of a new type or with altered characteristics can be a warning about impending TIA. But is headache a useful warning symptom? In other words, should such a headache lead to vascular work-up? In our opinion the answer is yes, under some circumstances. All middle aged to elderly patients who encounter a new type of headache should definitely have a vascular work-up. But also middle aged to elderly patients with a previously existing headache such as migraine or tension-type headache should be studied if the headache changes markedly in frequency or character despite the fact that the diagnosis is still the same. Special attention should be paid to patients with accelerating frequency of migraine that cannot otherwise be explained. Some may consider this a too aggressive attitude, but it must be remembered that diagnostic methods needed for prevention of cerebrovascular disorders are quite simple, pose no risk to the patient and that vascular episodes that can be prevented are often serious. Ultrasound examination of the neck vessels and blood tests may suffice, supplemented as necessary according to the degree of risk and other factors.

#### Anterior versus posterior circulation TIA

In agreement with previous studies in stroke and TIA [1–10], we confirm that headaches in TIA patients are more prevalent with posterior circulation TIA. In fact, all our patients with posterior circulation TIA had headache within the week before TIA and many at the time of TIA. The reason for this remains unclear but it is noteworthy that the interest in brain stem and hypothalamic mechanisms of migraine is increasing. During attack of migraine without aura, blood flow was increased in a small area of the ventro-medial medulla and this finding has later been confirmed [16, 17]. The vast majority of migraine auras are caused by blood flow changes thought to be caused by cortical spreading depression in the occipital cortex [18] and, finally, white matter abnormalities in migraine patients are primarily seen in the posterior fossa circulatory territory [15]. There are also reports of small pathologies in the brain stem associated with headache or migraine [17].

### Strengths and weaknesses of the present study

To the best of our knowledge this is the first study that has examined headache in TIA patients using a professional, face to face, semistructured interview describing all relevant characteristics of headaches associated with TIA.

One limitation of this study is the quick disappearance of clinical symptoms in TIA patients before admission to the hospital. Some of them could not remember details which are important in the differential diagnosis of TIA and MA. For example, some patients could have missed gradual spread of symptoms or presence of succession of symptoms. Some patients could not describe the characteristics of headache during TIA very well. Therefore some cases of MA could have been missed. However, we performed follow-up of 118 out of 120 patients with TIA. The period of follow-up varied from 6 months till 4 years. We found only one case of migraine with aura which was missed during the first interview. This patient experienced three more similar attacks of migraine with aura during two following years.

The weakness of the present study was using only CT in 8 patients which is not an accurate method for detection of small infarcts, especially in the posterior territory. However we performed a follow-up of these 8 patients during 3 years and nobody from them had recurrent episodes and other neurological problems. Also, the inaccuracy of patient’s recall of headache during the last year may have been a limiting factor.

## Conclusions

The one year prevalence of migraine was significantly higher in TIA patients than in controls and so was the prevalence of headache within the last week before TIA and at the time of TIA. A previous headache that worsens and a new type of headache can be a warning of impending TIA.

## Abbreviations

CT: Computed tomography
DWI: Diffusion weighted imaging
MRI: Magnetic resonance imaging
TIA: Transient ischemic attack
OR: Odd ratio
CI: Confidence interval
ICHD-3: International Classification of Headache Disorders
TTH: Tension-type headache
